# Epigenetic Modulation with HDAC Inhibitor CG200745 Induces Anti-Proliferation in Non-Small Cell Lung Cancer Cells

**DOI:** 10.1371/journal.pone.0119379

**Published:** 2015-03-17

**Authors:** Sung-Min Chun, Ji-Young Lee, Jene Choi, Je-Hwan Lee, Jung Jin Hwang, Chung-Soo Kim, Young-Ah Suh, Se Jin Jang

**Affiliations:** 1 Department of Pathology, Asan Medical Center, The University of Ulsan College of Medicine, Seoul, Republic of Korea; 2 Asan Institute for Life Science, Asan Medical Center, The University of Ulsan College of Medicine, Seoul, Republic of Korea; 3 Department of Oncology, Asan Medical Center, The University of Ulsan College of Medicine, Seoul, Republic of Korea; 4 Department of Urology, Asan Medical Center, The University of Ulsan College of Medicine, Seoul, Republic of Korea; Peking University Health Science Center, CHINA

## Abstract

Histone modification plays a pivotal role on gene regulation, as regarded as global epigenetic markers, especially in tumor related genes. Hence, chemical approaches targeting histone-modifying enzymes have emerged onto the main stage of anticancer drug discovery. Here, we investigated the therapeutic potentials and mechanistic roles of the recently developed histone deacetylase inhibitor, CG200745, in non-small cell lung cancer cells. Treatment with CG200745 increased the global level of histone acetylation, resulting in the inhibition of cell proliferation. ChIP-on-chip analysis with an H4K16ac antibody showed altered H4K16 acetylation on genes critical for cell growth inhibition, although decreased at the transcription start site of a subset of genes. Altered H4K16ac was associated with changes in mRNA expression of the corresponding genes, which were further validated in quantitative RT-PCR and western blotting assays. Our results demonstrated that CG200745 causes NSCLC cell growth inhibition through epigenetic modification of critical genes in cancer cell survival, providing pivotal clues as a promising chemotherapeutics against lung cancer.

## Introduction

Epigenetic modifications such as CpG DNA methylation or histone acetylation are regarded as an important step in cancer development and therefore have been studied to discover cancer biomarkers and therapeutic stratege [1–3]. Once cytosine methylation occurs on CpG dinucleotides via the action of DNA methyl transferase (DNMT), the methyl cytosine is maintained to the next generation due to the lack of a DNA de-methyl transferase in mammals. The irreversible histone modification has been also used as a biomarker for the early diagnosis or prognosis of cancer, as well as an effective target in cancer therapeutics [4,5]. Acetylation or methylation on lysine residues of H3 and H4 amino terminal tails are dominant histone modifications, and each is responsible for the expression of bound genes. For example, methylations on lysine 4 of H3 and lysine 27 of H3 are known as transcriptional activating and repressing events for histone bound genes, respectively. Histone acetylation on lysine 16 of H4 is related to transcriptional activation and/or replication initiation of corresponding genes. In normal cells, histone acetylation is precisely controlled by histone acetyl transferase (HAT) and histone deacetylase (HDAC). Hyper-acetylation of oncogenes or hypo-acetylation of tumor suppressor genes, however, is frequently observed in various cancers. HDAC inhibitors (HDACi) are the most developed anti-cancer drugs targeting epigenetic modulation and are being applied for the treatment of various cancers, particularly in solid tumors, such as breast, colon, lung, and ovarian cancers, as well as in haematological tumors, such as lymphoma, leukemia, and myeloma [6–9]. In addition, epigenetic dysregulation in lung cancer is often related with the overexpression of HDAC1 and aberrant methylation of certain genes, resulting in therapeutic efficacy of combination epigenetic therapy targeting DNA methylation and histone deacetylation. HDACs comprise three classes: Class I, HDAC 1, 2, 3, and 8; Class II, HDAC 4, 5, 6, 7, 9, and 10; and Class III, HDAC 11 (sirtuins 1–7) [10,11]. HDACi, trichostatin A (TSA) [12,13] or vorinostat (SAHA)[14–16] inhibit class I and II HDAC enzymes, resulting in growth arrest, apoptosis, differentiation, and anti-angiogenesis of cancer cells, when used independently or in combination with other anti-cancer agents. Mechanistically, the restoration of silenced tumor suppressor genes or suppression of activated oncogenes in cancer cells plays a critical role in the anti-cancer effects of drugs. This is followed by the induction of cell cycle arrest at the G1 stage through the expression of p21 and p27 proteins, or a G2/M transition delay through the transcriptional downregulation of cyclin B1, plk1, and survivin.

HDAC inhibitor CG200745, (E)-N(1)-(3-(dimethylamino)propyl)-N(8)-hydroxy-2-((naphthalene-1-loxy)methyl)oct-2-enediamide, has been recently developed and presently undergoing a phase I clinical trial. Its inhibitory effect on cell growth has been demonstrated in several types of cancer cells, including prostate cancer, renal cell carcinoma, and RKO cells (colon carcinoma cells) in mono- and combinational-therapy with other anticancer drugs [17–19]. The mechanism underlying the cell growth inhibition of CG200745 in RKO cells has been shown to occur in a p53-dependent manner [19]. Importantly, CG200745 increased acetylation of p53 at lysine residues K320, K373, and K382. CG200745 also induced the accumulation of p53, promoted p53-dependent transactivation, and enhanced the expression of proteins encoded by p53 target genes, *MDM2* and *p21* (Waf1/Cip1) in human prostate cancer cells. In current study, we evaluated the antitumor effects and explored the direct targets of a CG200745 on non-small cell lung cancer (NSCLC) cells to verify additional cancer indication. We analyzed cell proliferation and altered gene expression pattern upon histone deacetylation through ChIP-on-chip assay, real-time PCR quantification and western blotting. Our results suggest that the HDAC inhibitor CG200745 causes epigenetic reactivation of critical genes that are transcriptionally suppressed in cancers, and therefore can be a promising NSCLC cancer therapeutic.

## Materials and Methods

### Chemicals and cell lines

The HDAC inhibitors (HDACi), suberoylanilide hydroamic (vorinostat, SAHA) and CG200745, were provided by Crystal Genomics Co. (Seoul, Rep. Korea). These compounds were dissolved in DMSO and stored at -20°C until use. Human non-small cell lung cancer (NSCLC) cell lines and an immortalized normal bronchial epithelial cell line (Beas-2B) were purchased from American Type Culture Collection (Rockville, MD). All cell lines were cultured in RPMI 1640 media supplemented with 10% fetal bovine serum, 100U/mL penicillin, and 100μg/mL streptomycin with 5% CO_2_ at 37°C.

### Western blotting

50μg of whole cell extracts were run on SDS-PAGE gels and transferred onto PVDF membranes. The membranes were blocked and incubated with specific primary antibodies against H4K16ac, CCND1, p21, and caspase 3 (Cell Signaling, Beverly, MA), anti-H4S1/K5/K8/K12Ac from Santa Cruz (Santa Cruz, CA) and β-Actin from Sigma (St. Louis, MO). After incubation with appropriate horseradish peroxidase-conjugated secondary antibodies, bands were detected using ECL reagent (Amersham-GE Healthcare Life Sciences, Pittsburgh, PA).

### Real-time quantitative PCR

Total RNA preparation and cDNA synthesis were performed using TRIzol reagent (Invitrogen, Carlsbad, CA), and SuperScript III First-Strand Synthesis SuperMix (Invitrogen). Quantitative real-time PCR was carried out using Power SYBR Green PCR Master Mix (Applied Biosystems, Grand Island, NY) on IQ5 Multicolor Real-Time detection system (Bio-Rad) with each of forward and reverse primer for *CCNA2*, *CCNB1*, *CCND1*, *CCNE2*, *CDK2*, *Bax-a*, *bcl-2*, *p16*, *p21*, *p27*, and *Mxi1* (Bionics, Seoul, Korea) (S1 Table).

### Cytotoxicity assay

The IC_50_ of each HDACi on lung cancer cells was determined through cell proliferation assay using CellTiter 96 AQ_ueous_ One Solution Reagent (Promega, Madison, WI) according to the manufacturer’s protocol. After different time period incubation with HDACis, the absorbance was then measured at 490 nm on the Wallac 1420 Victor3 with WorkOut 2 software.

### FACS assay

The effect of CG200745 on NSCLC cell cycle was analyzed on Flow cytometry using propidium iodide (PI) staining. After incubation with CG200745 or DMSO, cells were fixed with ice-cold 70% ethanol at 4°C overnight. Genomic DNA was stained with 30μg/mL PI and the fluorescence was measured with a FACS Calibur machine (Becton—Dickinson, Mountain View, CA). The red fluorescence due to PI-stained DNA was collected at 560 nm dichroic mirror and a 600 nm pass filter (bandwidth, 35 μm). A total of 10,000 cells were collected by FACS and analyzed using the Cell Quest 3.1 software (Becton Dickinson).

### Chromatin immunoprecipitation (ChIP)

The ChIP assays were performed according to the manufacturer’s recommendations. Briefly, cells treated with 3μM CG200745 or DMSO for 24 h were precipitated with an anti-acetyl-H4K16 antibody (Upstate Biotechnology, Lake Placid, NY). After crosslinking with 1% formaldehyde, chromatin was fragmented into 300~800 bp size by sonication in Bioruptor, UCD-200 (Diagenode, Denville. NJ). Chromatin/protein complexes were precipitated with a modified histone specific antibody and Protein A/G plus immobilized Agarose beads. After elution from the beads, precipitated DNA-proteins were reverse-crosslinked, and DNA was purified with a PCR purification kit (Qiagen, Valencia, CA).

### Microarray experiments on ChIP products: ChIP-on-chip experiments

ChIP-on-chip assay was performed on an Agilent 244k × 2 oligonucleotide microarray as the manufacturer’s instruction (Agilent Mammalian ChIP-on-chip Protocol v.10). Briefly, the input control for whole cell extracts and the purified ChIP products were amplified by ligation-mediated PCR using an artificial oligonucleotide linker, after blunting the DNA ends using T4 DNA polymerase. Then, amplified input and ChIP products were labeled with fluorescent BioPrime Total Genomic Labeling System (Invitrogen). The same amount of input DNA and ChIP product with different fluorescent dyes were mixed with human Cot-1 DNA (1 mg/mL), and hybridized onto an Agilent 488k promoter array (two 244k arrays), containing ~17,000 of the defined human transcripts covering from -5.5 kb to +2.5 kb of transcription start site (TSS). Hybridized images were visualized with the microarray scanner (Revolution 4200; Vidar Systems Cor., Herndon, VA).

### Data Analysis and Ontology analysis

Histon acetylation of ChIP products and input was analyzed using the ChIP module of the DNA Analytics (Agilent, version 4.0.81) with Tukey biweight normalization, whitehead error model, and whitehead Per-Array Neighborhood model. Briefly, log2 ratio for each gene of CHIP products and input was calculated on the average of probes in 1,000bp, considering genes with a P value of lower than 0.1 as valuable. In order to depict H4K16ac status from the transcription start site (TSS), the distance for each probe was simplified as follows: region between-1,000~ -500 from the TSS was numbered as-2, -500~TSS as-1, and TSS~+500 as +1, as the TSS position defined from-11 to +10.

Functional annotations through quantitative RT-PCR and western blotting were followed by the analysis using the DAVID (Database for Annotation, Visualization, and Integrated Discovery) bioinformatics resource and Gene Ontologies (Molecular Function, Biological Process, Cell Components) for signaling pathways. All categories representing less than 4% of the total number of applied genes were excluded.

## Results

### CG200745 inhibits the proliferation of NSCLC cells

**Fig 1:**
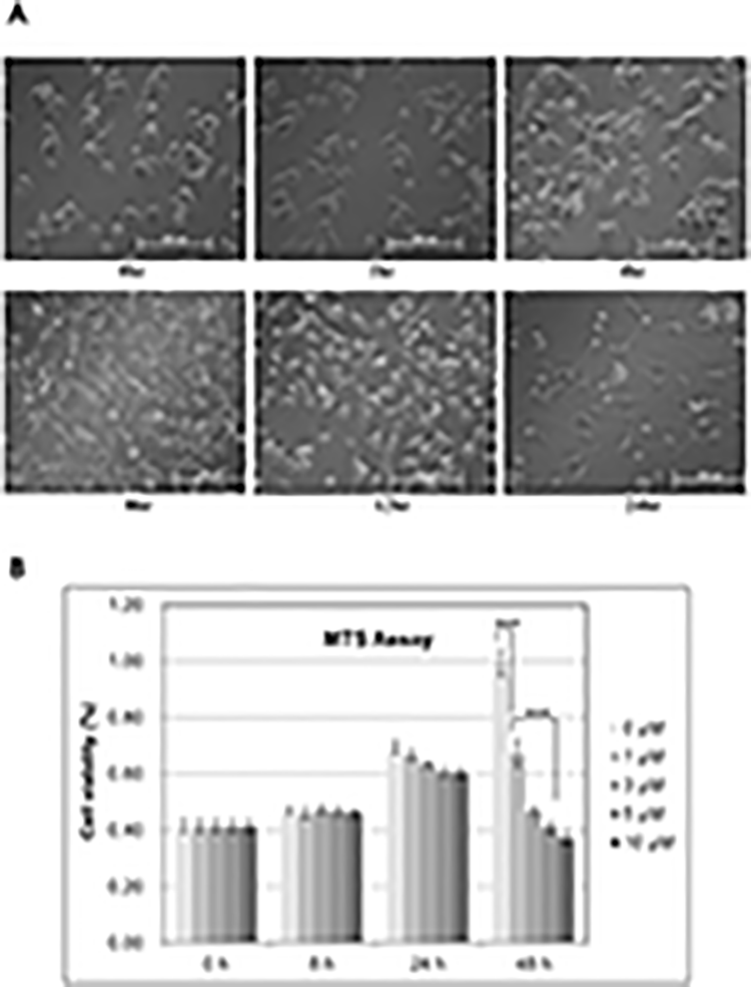
Anti-proliferation effect of CG200745 on Calu6 cells. Calu6 cells grown on 6-well plates were treated with 3 μM CG200745 for the indicated time periods to analyze the anti-proliferation effect of CG200745, and were observed under microscope. (B) The growth of Calu6 cells in 96-well plates was analyzed via MTS assay upon treatment with various concentrations of CG200745 (0 to 10μM) in time-course (0 to 48 hours). Statistical significance of changes in cell viability was calculated using t-test (*** indicates p<0.001).

To determine the inhibitory effects of CG200745 on cell proliferation, we measured the IC_50_ of CG200745 of the following lung cancer cell lines: Calu6, H358, H596, A549, HOP62, HOP92, H226, H460, and H522, as well as Beas2B cells. IC_50_ values of CG200745 in the tested lung cancer cells were at micromolar levels, and were comparable to those obtained from the reference drug, SAHA (Table 1), indicating a significant growth suppression effect of CG200745 on various NSCLC cell lines. Among them, A549, Calu6, HOP92, and H522 cells were the most sensitive with IC_50_ of <3 μM. The effect of CG200745 on Calu6 cell proliferation was confirmed as the cell number and morphology on optical microscopy after exposure to 3 μM CG200745 at different time periods (Fig. 1). Calu6 cells showed regular cell growing patterns in a time-dependent manner until 8 hours after drug treatment. However, after 8 hours of CG200745 treatment, the growth rate of Calu6 cells was reduced, and cell death with morphological changes was noted. The adverse effect of CG200745 on Calu6 cell growth was analyzed by performing MTT assay with various conditions, confirming that the drug reduced the cell proliferation to 40% of untreated cells (Fig. 1A).

**Table 1:** IC50 value for SAHA and CG5 on various lung cancer cells.

Drug	Beas2B	Calu6	H358	H596	A549	HOP62	HOP92	H226	H460	H522
SAHA	0.37	3	4.3	7.2	1.5	1	3	8	2.6	2.1
CG5	0.51	2.8	8.1	8.6	2.1	3.3	2.1	5	3.8	2.2

### CG200745 treatment induces G2/M cell cycle arrest and apoptosis in Calu6 cells

**Fig 2:**
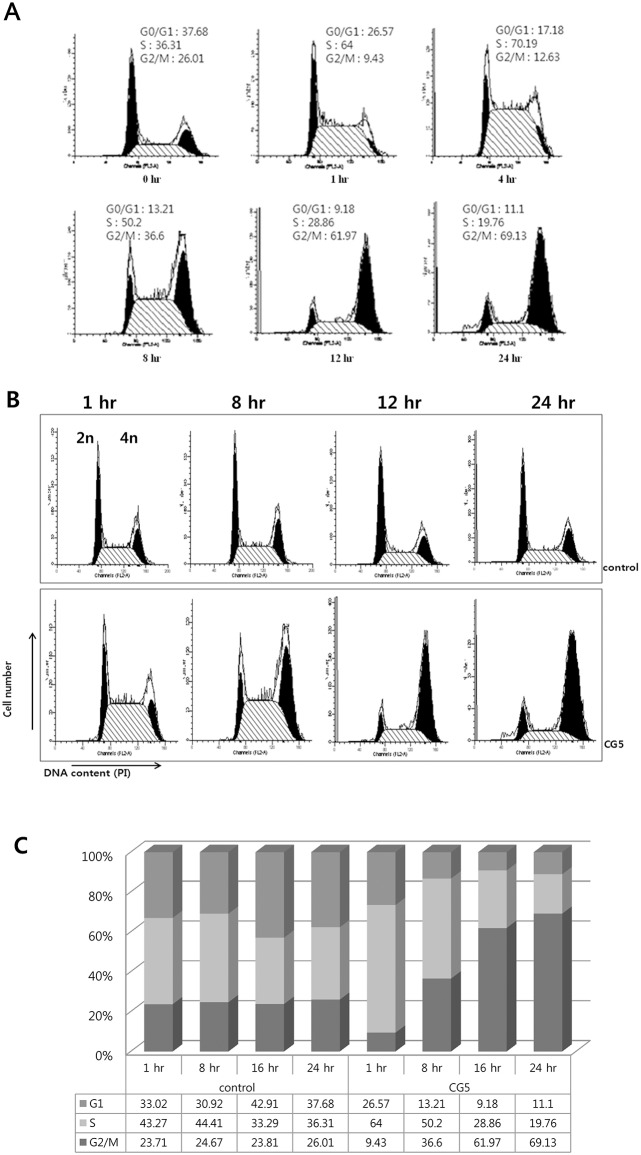
Flow cytometric investigations of CG200745-treated Calu6 cells. Calu6 cells treated with 3 μM of CG200745 were analyzed cell cycles on flow cytometer. Each histogram consists of two black peaks and one scratched region indicating G0/G1 phase, G2/M phase, and S phase, respectively. The effect of CG200745 on the cell cycle distribution was analyzed at for various time points (A), or compared to untreated cells (B) depicting as a graphical view (C).

HDACi compounds are known to induce cell cycle arrest, an important cancer target mechanism, in various cancer cells. To examine whether CG200745 induced cycle arrest in the Calu6 cell line, these cells were treated with this HDACi molecule for various time points from 0 to 24 hours, followed by flow cytometric analysis (FACS). As shown in Fig. 2A, an increase in the G2/M population was observed in a time-dependent manner. CG200745-treated Calu6 cells showed a significantly increased cell proportion in G2/M phase (69%) compared to untreated control cells (26%) (Figs. 2B and 2C). These results indicate that CG200745 causes cell growth inhibition through G2/M phase cell cycle arrest.

### CG200745 causes increased histone acetylation

**Fig 3:**
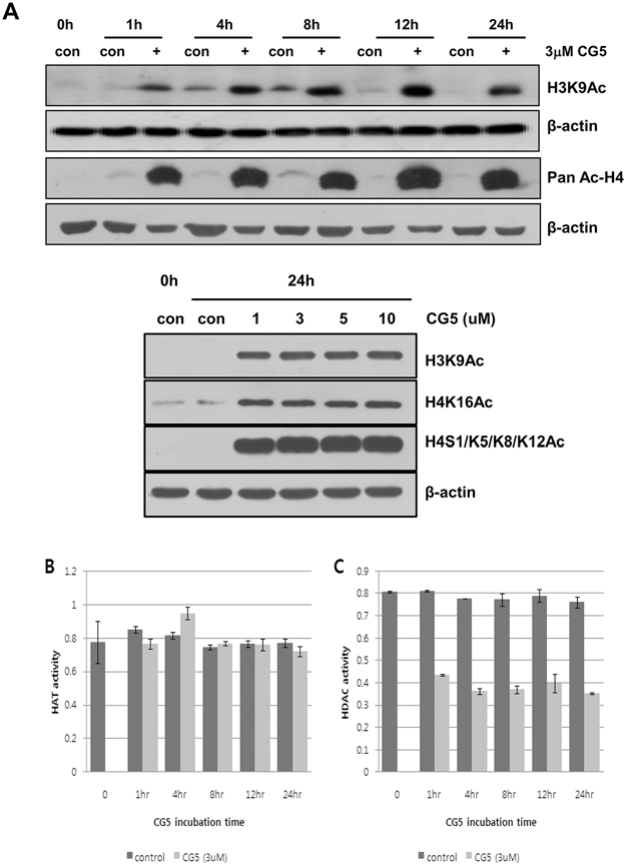
The effect of CG200745 on histone acetylation in Calu6 cells. **(A)** Acetylation status of histones were analyzed on Western blotting of Calu6 cells treated with various concentrations of CG200745 (0 to 10 μM) with anti-H3K9ac, anti-H4K16Ac, anti-H4S1/K5/K8/K12Ac and/or anti-acetyl H4 antibodies. (B, C) HAT and HDAC activities in control and CG200745-treated cells were determined using 50μg of nuclear extract at indicated time point.

To examine the HDAC inhibitory effects of CG200745 on non-small cell lung cancer cells, we treated Calu6 cells with different concentrations of CG200745 and analyzed histone acetylation by western blotting, as well as HAT and HDAC activity at various time points. As shown in Fig. 3A, low concentration of CG200745 treatment significantly increased the acetylation of histone H3 and H4 at various sites including K9 on H3, and K16 as well as S1/K5/K8/K12 on H4, in a time-dependent manner up to 24 hours after treatment in western blotting analysis. To confirm the inhibitory effects of CG200745 on histone deacetylation, we measured HAT and HDAC activity in nuclear extracts of Calu6 cells before and after CG200745 treatment. In contrast to the unchanged HAT activity after CG200745 treatment (Fig. 3B), HDAC activity in CG200745-treated Calu6 cells was significantly lower than it in untreated control cells (Fig. 3C), indicating that CG200745 increased histone acetylation level due to the inhibition of HDAC activity.

### CG200745 induces histone modification changes in Calu6 cells

H4K16ac is a well-known activator of global gene transcription. To investigate whether genes regulating cell survival were affected by histone acetylation resulting from CG200745 treatment, we performed ChIP-on-chip assay using an H4K16ac specific antibody. Genes bound to H4K16ac were immunoprecipitated and analyzed on oligonucleotide microarray focused at the promoter region. The main purpose of this experiment was to determine how many and which genes related to H4K16ac status are affected by CG200745 treatment. As shown in Table 2, more than 10,000 genes bound to H4K16ac were detected in both control- and CG200745-treated cells, with the expression levels of thousands of genes showing changes on the H4K16ac ChIP-on-chip assay after CG200745 treatment.

**Table 2:** H4K16ac modified genes.

H4K16ac	ChIP/input (log ratio)	# of genes
**Control**	Control >= 0.6	14,024
**CG5 treated**	CG5 >= 0.6	10,783
**Control & CG5**	Control & CG5 >= 0.6	5,916
**Increased by CG5**	CG5-control >= 0.6 & CG5 >= 0.6	3,199
**Decreased by CG5**	CG5-control <= -0.8 & Control >= 0.6	4,158
Increased by CG5*	CG5-control >= 0.6 & CG5 >= 0.6around TSS (-2~2)	494
Decreased by CG5*	CG5-control <= -0.8 & Control >= 0.6around TSS (-2~2)	2,669

Total numbers of genes which are modified with H4K16ac were assessed by ChIP-on-chip analysis in CG5 treated and un-treated control calu6 cells. Log ratio of ChIP versus input was used for calculating H4K16ac status, and genes with more than 0.6 were considered as immuno precipitated products.

**Fig 4:**
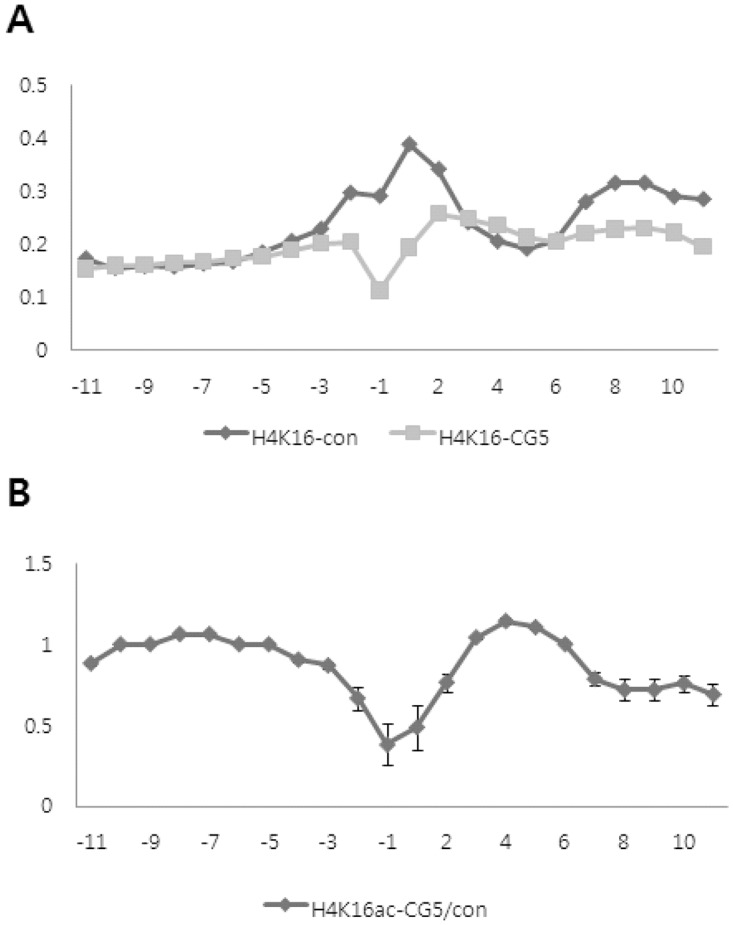
H4K16 acetylation status adjacent to transcription start site (TSS). H4K16 acetylation adjacent to the TSS was assessed by calculating the average ChIP/input log ratio at the same distance from the TSS. The numbers in the X axis indicate the distance from TSS. Y axis represents the average ChIP/input log ratio. (A) H4K16 acetylation status of genes around TSS was individually depicted in CG200745-treated or untreated Calu6 cells. (B) The level of H4K16 acetylation was compared between CG200745-treated and untreated cells according to the distance from the TSS. Two separate experiments were performed to calculate the error bar for each position.

We then examined the pattern of H4K16ac changes according to the distance from the TSS by plotting an average log value of ChIP/input for all probes (Fig. 4), in which the X axis denoted the distance from the TSS area as described in a previous section. The H4K16ac was, surprisingly, decreased at the adjacent area to the TSS, especially at-500 nt from the TSS, after CG200745 treatment, whereas H4K16ac at distant area from TSS was no difference with it in untreated control cells, which is common due to the transcriptional activation function of H4K16 (Fig. 4A). The average log ratio of ChIP/input data clearly demonstrated that fewer genes with H4K16ac at 500 nt from the TSS were seen in treated cells compared to control cells, although a major modification site for H4K16ac was still evident around the TSS (Fig. 4B). These results were confirmed in repeated experiments, indicating that CG200745 treatment suppresses H4K16 acetylation at 500 nt from the TSS of many genes.

### Correlation between changes in H4K16ac and gene expression following CG200745 treatment

H4K16ac-induced gene expression is driven by the loosening of chromosomal DNA bound to histone proteins, resulting in the opening of DNA sites for various transcription factors. Under a hypo-acetylated status, however, the positive charge of the lysine residues on the amino terminus of the histone tail acts as a source for high affinity binding to the negative charge of the phosphate backbone in chromosomal DNA. This tight binding allows chromatin to form more compact structures, resulting in the inhibition of transcription of related genes. As shown in Table 2, the H4K16ac levels of thousands of genes were changed after CG200745 treatment. In order to verify the correlation between H4K16ac status and corresponding gene expression, mRNA levels were examined for genes that underwent significant H4K16ac changes around the TSS area. As shown in Table 3, the mRNA expression levels of *p*21, *GSN-b*, and *Mxi1*, which showed increased H4K16ac, were all increased after CG200745 treatment, whereas other genes with decreased H4K16ac, such as *HOXD11*, *HOXD9*, and *PIM1*, showed decreased mRNA expression. In addition, the mRNA expression of *HAT1*, which showed similar H4K16ac levels with and without CG200745 treatment, was not changed.

**Table 3:** Correlation between H4K16ac and mRNA expression.

	ChIP-on-chip data: H4K16ac			RQ_PCR data		
Genes	Control	CG5(3uM, 24hr)	CG5/con	Control (Ct)	CG5(Ct)	ΔΔCt
B2M	0.56	0.25	0.44	14.91	15.95	0.00
CCNA2	0.59	0.32	0.55	20.84	22.13	0.25
CCNB1	0.44	0.35	0.80	18.58	19.09	**-0.53**
CCND1	0.77	0.30	0.39	18.03	19.32	0.25
CCNE1	0.45	0.21	0.47	20.78	21.98	0.16
CDKN1A(p21)	0.23	0.31	**1.37**	27.31	25.68	**-2.67**
CDKN1B(p27)	0.73	0.17	0.23	19.13	20.77	0.60
DNMT1	0.58	-0.04	-0.06	22.10	23.80	0.66
ENO1	0.81	0.14	0.17	17.21	18.98	0.73
GSN-b	0.47	0.67	**1.42**	22.01	22.21	**-0.84**
HAT1	0.42	0.40	0.95	20.02	21.01	-0.05
HOXD11	0.41	0.35	0.86	23.09	26.47	2.34
HOXD9	0.50	0.26	0.53	25.35	29.12	2.73
MXI1	0.01	0.20	**15.96**	29.69	27.49	**-3.24**
MYST2	0.59	-0.10	-0.17	23.10	24.91	0.77
PIM1	0.40	0.18	0.45	22.87	26.68	2.77
TOP2B	0.37	0.26	0.70	22.65	23.91	0.22

**Fig 5:**
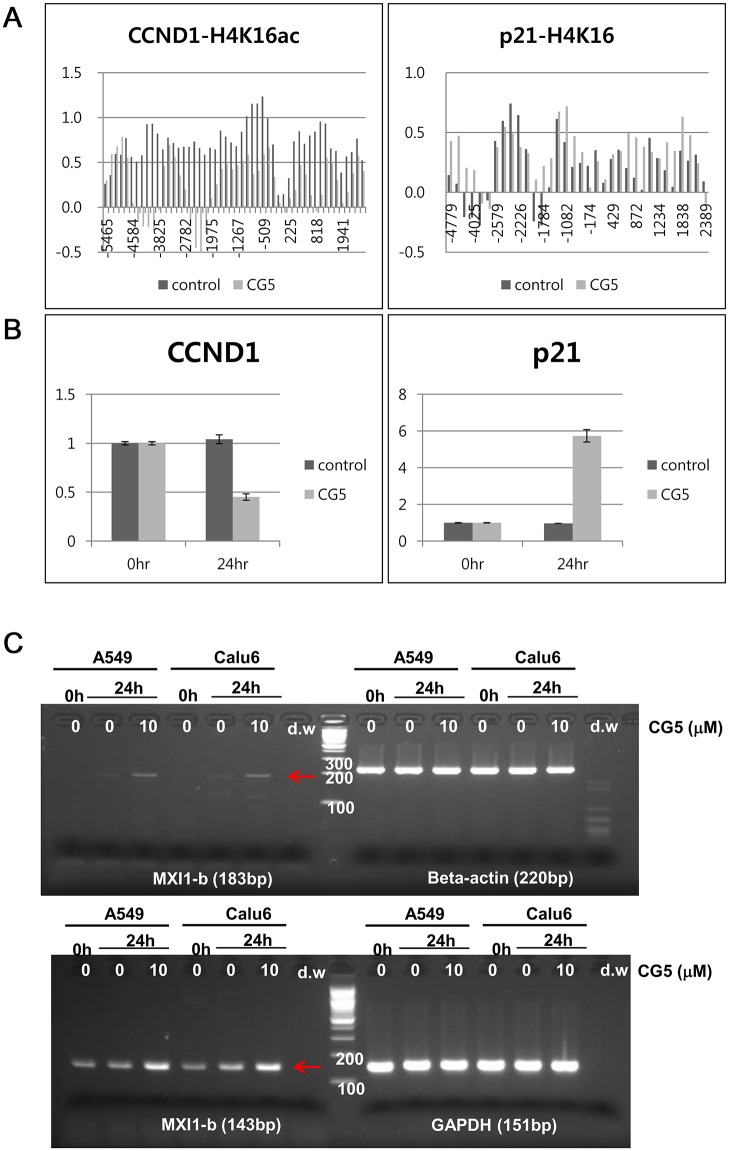
Correlation between H4K16ac and gene expression. **(A)** The levels of H4K16 acetylation on CCND1 and *p*21 genes were denoted according to the distance from the TSS in untreated (control) and CG200745-treated cells (CG5). (B) The mRNA levels of the CCND1 and *p*21 genes were quantified by real-time RT-PCR. (C) The expression of the Mxi1 gene was determined by RT-PCR in A549 and Calu6 cells.

**Fig 6:**
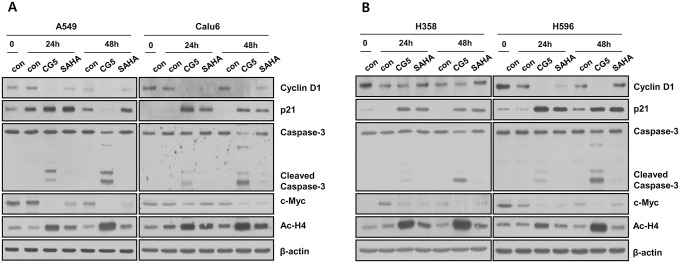
Changes in protein expression of NSCLC cells were analyzed to evaluate the effect of CG200745 or SAHA for 24 and 48 h. Cells were analyzed on Western blotting with cyclin D1, p21, caspase-3, c-myc, acetyl-H4, H3, and β-actin antibodies. Cell lysates of drug-treated A549, Calu6 (A), H358, and H596 (B) cells were compared to corresponding untreated cells.

To further confirm the correlation of mRNA expression and changes in H4K16ac after CG200745 treatment, mRNA expression of the *CCND1* and *p21* genes were assessed. The H4K16ac levels of the *CCND1* and *p21* -promoter region were analyzed in untreated (control) and CG200745-treated (CG5) Calu6 cells using a whole gene probe microarray. As shown in Fig. 5, the H4K16ac level in *CCND1* and *p21* genes after CG200745 treatment, especially near the TSS position, was found to be significantly altered (Fig. 5A) and to be correlated with the expression of mRNA when quantitatively measured (Fig. 5B). Expression of *Mxi1* was increased after 24 hours treatment of CG200745 on Semi quantitative analysis with two different sets of primers (Fig. 5C). The protein expression levels of *cyclin D1* and *p21* genes were dramatically reduced and increased, respectively, not only in Calu6 cells but also in several other NSCLC cells including A549, H358, and H596 cells (Fig. 6). In addition, CG200745 treatment resulted in activation of the intrinsic apoptotic caspase 3, in Calu6 cells as well as in several NSCLC cells including A549, H358, and H596 cells (Fig. 6A and 6B). Taken together, these data showed that the expression levels of certain genes were affected by CG200745 treatment through the H4K16ac level at the TSS region.

## Discussion

HDACi molecules have been reported to induce a range of anticancer effects, including tumor cell apoptosis, cell cycle arrest, differentiation, senescence, modulation of immune responses, and altered angiogenesis [20]. Furthermore, the use of HDACi has been shown to increase the sensitivity of NSCLC cell lines to gefitinib or erlotinib [21,22]. CG200745, (E)-N(1)-(3-(dimethylamino)propyl)-N(8)-hydroxy-2-((naphthalene-1-loxy)methyl)oct-2-enediamide, is a recently developed HDACi, presently undergoing a phase I clinical trial. Its inhibitory effect on cell growth has been demonstrated in several types of cancer cells, including prostate cancer, renal cell carcinoma, and RKO cells (colon carcinoma cells) in mono- and combinational-therapy with other anticancer drugs [17–19]. This drug in combination with Docetaxel induced tumor regression in DU145 prostate cancer cell xenograft via activation of apoptosis. Also, it caused cell death in colon carcinoma cells expressing wild type p53 by acetylation of p53. In our current study, we investigated the antitumor effects of HDACi on NSCLC cells to potentially expand its clinical application with broad tumor indications. We further intended to elucidate the underlying mechanism of the novel HDAC inhibitor which is now in clinical trials for leukemia and solid tumor, by which proper clinical application would be achieved, such as co-treatment and/or adjuvant treatment with targeted chemotherapeutics. HDACi treatment induced the inhibition of lung cancer cell growth, cell growth arrest at G2/M phase, and apoptosis evidenced by caspase 3 cleavage at comparable IC_50_ concentrations to that of SAHA (also known as Vorinostat), the only HDACi currently approved for the clinical treatment of cutaneous T-cell lymphoma [23–25]. Although H4K16 acetylation still occurred broadly in promoter regions, interestingly, CG200745 specifically decreased histone H4K16 acetylation at-500 nucleotides from the TSS as shown by repeat CHIP-on chip analysis. These results indicate that HDAC inhibitor-mediated cell growth inhibition involves the transcriptional regulation of multiple genes. In-depth analysis of genes involved in cell cycle regulation, such as *CCND1*, *p21*, and *Mxi1*, showed that CG200745 treatment results in transcriptional changes in these genes.

*Mxi1*, an antagonist of the *c-Myc* oncogene, encodes a protein designated Mad2, which is a transcription factor in the Myc-Max-Mad network. The Mad/Mxi heterodimer is predicted to antagonize Myc function by abolishing available Max for myc transcriptional activity. The transcription factor c-Myc is overexpressed in many human tumors, and its activation requires heterodimerization with partner Max. C-myc is regulated through ubiquitination and proteasome degradation [26,27]. In fact, inhibition of Myc/Max binding by small molecules has been identified as a highly promising target for cancer therapy [28]. Mxi1-related proliferation is mediated through IGFBP-3 signaling [18], and increased FoxO3a expression upregulates the expression of members of the Mad/Mxi pathway [29]. Upregulated expression of Mxi1 by CG200745 treatment may therefore play a role in the regulation of cell proliferation via the Myc-Max-Mad network.

The SAHA HDACi, has been shown to decrease the expression levels of Erk1/2 and MMP-9, increase the expression levels of p53, which in turn alters cell proliferation and apoptosis, and promote the acetylation of histones H3 and H4 in ovarian carcinoma cells [30]. Furthermore, a previous study has reported that SAHA activates p53, but does not require p53 for its anticancer effects. The same study showed that entinostat-induced cytotoxic effects are partially dependent on p53, indicating that different HDACi compounds have different requirements for p53 [31]. The mechanism underlying the cell growth inhibition of CG200745 in RKO cells has been shown to occur in a p53-dependent manner [19]. In that study, CG200745 increased acetylation of p53 at lysine residues K320, K373, and K382. CG200745 also induced the accumulation of p53, promoted p53-dependent transactivation, and enhanced the expression of proteins encoded by p53 target genes, *MDM2* and *p21* (Waf1/Cip1) in human prostate cancer cells. The antitumor effects of CG200745 on NSCLC cells, however, were found in our current analyses to be independent of mutation status of the genes encoding p53. The NSCLC cells we used, including p53 wild type and null/mutant cells, showed cell growth inhibition upon treatment with an HDACi, indicating that CG200745 regulates cell growth by different mechanisms according to cell context.

In conclusion, findings of our present study expand the general applicability of CG200745 to the treatment of NSCLC, for which it is a promising new HDACi. In addition, our results demonstrate that the effects of this HDACi may occur via a p53-independent mechanism. Furthermore, the data shows that HDACi may suppress the c-myc signaling pathway through the induction of *Mxi1*, but this finding requires further investigation. Finally, the potential of CG200745 to enhance the potency of anticancer drugs as part of a combination therapy should be further explored

## Supporting Information

S1 Table Primer sequences for quantitative RT-PCR reaction. (DOCX)
