# Supplementary material for: Epigenetic Modulation with HDAC Inhibitor CG200745 Induces Anti-Proliferation in Non-Small Cell Lung Cancer Cells
Source: PLoS One. 2015 Mar 17;10(3):e0119379. doi: 10.1371/journal.pone.0119379 (PMC4363698; doi:10.1371/journal.pone.0119379)
Supplement: S1 Table — (DOCX) [file pone.0119379.s001.docx]

|  | **Forward primer (5'-3')** | **Reverse primer (5'-3')** | **Product size (bp)** | **Tm** |
| --- | --- | --- | --- | --- |
| **B2M** | GTGCTCGCGCTACTCTCTCT | TCTGAATGCTCCACTTTTTCAA | 230 | 60/59 |
| **CCNA2** | GGTACTGAAGTCCGGGAACC | ATCCACATGAATGGTGAACG | 200 | 60/59 |
| **CCNB1** | CGGGAAGTCACTGGAAACAT | AAACATGGCAGTGACACCAA | 220 | 59/60 |
| **CCND1** | CGTGGCCTCTAAGATGAAGG | CTGGCATTTTGGAGAGGAAG | 220 | 59/59 |
| **CCNE1** | GGGACACCATGAAGGAGGAC | TCGATTTTGGCCATTTCTTC | 150 | 61/60 |
| **CDKN1A(p21)** | GGAAGACCATGTGGACCTGT | TAGGGCTTCCTCTTGGAGAA | 179 | 59/59 |
| **CDKN1B(p27)** | CATTTGGTGGACCCAAAGAC | GGGGAACCGTCTGAAACATT | 180 | 60/60 |
| **DNMT1** | CAGCTCGAGGACCTGGATAG | TTCCGGTAGTGCTCTGGGTA | 200 | 59/60 |
| **ENO1** | GTACCGCCACATCGCTGAC | CATGGCTTCCCTGAAGTTTG | 180 | 62/60 |
| **GSN-b** | CGACGCCTACGTCATCCT | CGGCCGTTCAGGTAGTCAT | 177 | 59/61 |
| **HAT1** | CCTACTCAGTTCTCAGTCCAACA | CCACGTCAATAAAGCTAGCAG | 166 | 58/57 |
| **HOXD11** | GGCAGTCCCTGCACCAAG | GGTATAGGGACAGCGCTTTTT | 190 | 62/59 |
| **HOXD9** | CTGTTCGCTGAAGGAGGAG | TTTCTCCAGCTCAAGCGTCT | 190 | 58/60 |
| **MXI1** | GCGAGAACTCGATGGAGAAG | GTGGGGGCTTTGAATGCT | 180 | 60/61 |
| **MYST2** | AGCCTACCCCAGTGACACC | GCATTTCCAGTTGGAGTTCG | 190 | 59/60 |
| **PIM1** | GAGGTTGGGATGCTCTTGTC | CTGAGTAGACCGAGCCGAAG | 180 | 59/60 |
| **TOP2B** | TGGGATGTTTGTCTCACATTG | GGTTTCACTGATACACCAGCTTT | 180 | 59/59 |

**Table S1. Primer sequences for quantitative RT-PCR reaction.**
